# Validation and analysis of the metric properties of the Leadership Virtues Questionnaire in work and organizational psychologists and individuals who perform leadership functions in Chile

**DOI:** 10.1371/journal.pone.0297906

**Published:** 2024-04-18

**Authors:** Pablo Livacic-Rojas, María José Rodríguez-Araneda

**Affiliations:** School of Psychology, University of Santiago of Chile, Santiago Metropolitan Region, Chile; UGR: Universidad de Granada, SPAIN

## Abstract

The literature on leadership and personal competencies exhibits limitations in terms of construct definition, behavior specifications and valid theory-based measuring strategies. An explanatory design with latent variables and the statistical software SAS 9.4 were used for the validation and adaptation to Spanish of the Leadership Virtues Questionnaire applied to work and organizational psychologists and people who exercise leadership functions in Chile. The levels of agreement between judges for the adaptation to the Spanish language and the confirmatory factor analysis of first order with four dimensions shows insufficient statistical indices for the absolute, comparative and parsimonious adjustments. However, a second-order confirmatory factor analysis with two dimensions presents a satisfactory fit for the item, model, and parameter matrices. The measurement of Virtuous Leadership would provide relevant inputs for further evaluation and training based on ethical competencies aimed at improving management, which would, in turn, allow for its treatment as an independent variable to generate an ethical organizational culture.

## Introduction

According to Dye [1], few authors can operationalize leadership despite the considerable increase in publications in recent years. According to this author, a main cause of this scenario in Psychology would be the conduction of research based on bibliographic reviews and the principles of leadership, but also addressing the topic in terms of management (control and results orientation) rather than evidence-based studies (processes oriented to organizational changes).

Since competencies are partially performed through covert behaviors (unobserved personal thoughts or attitudes) that conflict with social rules, they are hardly detectable from measurement instruments. In this sense, Regojo [2] indicated the importance of ethical competencies in the decision-making process.

Considering the social and economic environment in recent decades, Canals (in Echevarría [3]) believes that the changes in recent decades (e.g., technological innovations and financial deregulations due to economic volatility) amidst a "tsunami of digital transformation” ☯uncertainty, hyper-competitiveness and constant change (Forcaro, in Vilallonga [4]; Cardona and Rey [5])] have impacted talent management and the development of organizations.

Likewise, Regojo [2] states that the "insider trading" cases (almost 35 years ago) in different companies and organizations, triggered a need to combat immoral actions that directly and indirectly led decision-makers to solve problems of a technical nature (incentive design errors, administrative malfunctioning and incorrect risk analysis and management) as well as ethical behaviors (dishonest actions, negligence, procrastination, low responsibility in updating knowledge and skills) in government, direction and management.

Therefore, it is not surprising that organizations have given importance to moral behavior (Ewest [6]; Sison [7]) because, according to Pérez-Latre [8], in the last thirty years, the contexts of uncertainty (economy, pandemic, wars, media, social networks, technology, society and politics, among others) generated crises in trust, management, communication, the common good and leadership in different institutions. Therefore, according to data from the Edelman Barometer (in Pérez-Latre [8]), people today seek workplaces based on their "values and convictions", increasing the demands of social leadership in different institutions.

In this respect, in order to face immoral actions and lack of virtues, Lussier and Achua [9] have reported that organizations spend 2.2 trillion dollars in training and education and approximately 10,000 million in leadership. Furthermore, Hogan et al. (in Daniels and Daniels [10]) point out that the failure rate of leaders in competent management has fluctuated between 50% and 60%. Along this line, Cardona and Rey [5] believe that companies that add “a sense of purpose" (management that combines competency-based leadership with “mission-based management " tools) achieve better results (up to 240%) than those that do not, since they would articulate competencies (ethical, technical, managerial and interpersonal) with managerial excellence and the results associated with management (Regojo [2]). Furthermore, Luthans et al. [11] indicate that leading by example plus solid ethics and morality are among the most valued characteristics by followers.

The more than 66 existing leadership theories make progressive practice and research in a more convergent way difficult, as each of them provides a unique perspective, with the logical consequence of a lack of homogeneity among them (Yukl et al. [12]; Fuller et al. [13]; Ewest [6]).

Luthans et al. [11] classify the different groups of leadership theories into traditional (traits, states and development of abilities, groups and exchanges, leader-member exchange, contingency theory, path-goal) and modern (charismatic, transformational, authentic, substitutes, intercultural, focused on competencies, among others; see also Dye [1]; Cardona et al. [14]). More recently (associated with the pandemic and teleworking), these authors suggest that a new type of leadership has emerged from modern theories: the e-leader, who focuses on speed, technology, and takes high risks and high profits in short periods of time.

Based on modern theories and despite the vagueness of the term "style", Luthans et al. [11] have developed a leadership questionnaire from which three styles emerge, namely: autocratic (oriented towards high productivity), shared (high morale and productivity), and laissez-faire (high morale). In turn, different authors propose other models, such as full-range leadership measurable through the multifactorial leadership questionnaire (MLQ by Avolio et al. [15]), which estimates transformational, transactional, and laissez-faire styles.

At the same time, other authors have proposed other styles of leadership, namely bureaucratic, charismatic, relationship-oriented, task-oriented, servant leadership, adaptive (Kibbe [16]; Aasland et al. [17]; Kumar et al. [18]), ethical (based on virtues, Riggio et al. [19]), moral capital (Sisón [7]), ethical competencies (Regojo [2]), and destructive (Fosse et al. [20]).

Despite this high variability, different authors agree on the existence of a paradox that, on one hand, has a construct with an ambiguous definition (Ewest [6]) and, on the other hand, consists of easily observable behaviors (Reiche et al. [21]; Havard, [22]) in which a person influences other individuals by guiding, structuring, and facilitating the fulfillment of tasks and collective relationships (Reiche et al. [21]; Lussier et al. [9]). Furthermore, the accuracy of the construct requires considering that in leadership, the objective is for people to advance in the achievement of long-term goals, while in management the objective is to make things move forward (Havard [23]).

Perhaps one of the causes of this conceptual limitation is related to the use of intuitive approaches (Daniels et al. [10]; Daniels in Bailey [24]; Gadaire et al. [25]) or models that are not oriented to observational behavior (theories of personality or temperament) instead of evidence-based approaches to the development of leadership competencies that organizations would demand.

In an attempt to solve this situation, Daniels et al. [10] (based on the correlation of the laws of nature with human behavior) propose a model of four categories (moment, commitment, initiative and reciprocity) with three measurements each to record the responses of followers in the leadership measurement process. Specifically, based on impulse, the "moment" dimension proposes a system of behaviors in three measures: mass (perform more behaviors than those indicated in the job description or its management), speed (behavioral activation as a consequence of the absence of punitive contingencies) and direction (clarity in communication). In turn, based on the persistence of the behavior, the "commitment" dimension proposes a system of behaviors in three measures of the follower: vision (behavioral skills to stay focused over time), value (promote ethical behavior at all times) and persistence (behavioral effort that is oriented to reinforcing contingencies in the environment). For its part, based on individual behavior oriented towards cooperative behavior, the "initiative" dimension refers to a system of behaviors in three measures of the follower: teamwork (behavior supporting their co-workers), interfaces (behavior cooperation between different departments) and innovation (suggestions from followers to strengthen the mission and vision). Finally, based on the system of positive interpersonal behaviors, the "reciprocity" dimension proposes a system of behaviors in three measures of the follower: trust (sharing failures and errors with peers and leaders in order to benefit from them), respect (contact with the leader in search of advice or contributions for professional and personal decisions) and growth (encouraging and reinforcing leadership behaviors in followers). For this purpose, they have built a test and put forward a checklist for its registration, which aim to develop contingencies that favor the formation and maintenance of leadership.

For their part, Mattaini [26] state that effective communication, structuring reinforcement programs, and avoiding aversive control practices are among the essential practices of leadership.

Along a similar line, Cardona et al. [14] indicate the need to define and evaluate leadership based on competencies and with a model that operationalizes them through observable and habitual behaviors ☯see also Parks et al. [27]; Wilder et al. [28])]. In this context, Dye [1] points out that competency theories emphasize that leaders need to demonstrate broad behavioral repertoires in different fields, specifying around 80 critical competencies for effective leadership. A strength of this approach is that it provides a series of direct and observational indicators to measure competencies.

Along the same lines, Cardona et al. [14] believe that for this to be effective, the diagnosis of competencies should consider work needs in the different areas where the organization requires improvement. This pairing would enable the increase of management by purpose (organizational level, Cardona et al. [5]) and "internal success" (level of personal dimension, Sarráis [29]) or self-management of competencies (Lussier et al. [9]), which appear as relevant factors in a contingency organization system, leading to positive consequences for performance improvement, increased inclusion, well-being and mental health (Daniels et al. [30]). *Contra sensu*, a destructive leadership (Fosse et al. [20]) or *laissez-faire* style (Riggio et al. [19]) tends to generate environments characterized by organizations of aversive contingencies (discrimination and lack of inclusion) that negatively impact mental, physical, and spiritual health (APA [31]; Koenig [32]; Sarráis [29]).

In order to operationalize the observable behaviors proposed by different models of ethical leadership (or virtue-based leadership), Titus et al. [33] propose a comparative theoretical model in thirteen dimensions (performative, perfective, purpose, ethics, personal individuality, interpersonality, behavior models, moderating, preventive, non-reductionist, applied, vocational, openness and transcendence) based on the contributions of Saint Thomas Aquinas and Martín Seligman (Positive Psychology). In this regard, only in two dimensions (personal individuality and non-reductionist) would Positive Psychology provide more theoretical contributions than Thomism.

In particular, Riggio et al. [19] have focused on the operationalization of virtue-based ethical leadership, based on the behaviors of prudence, temperance, fortitude and justice (far from religion or spiritual connotations, despite proposing it from the works of Saint Thomas Aquinas). In this context, virtues would be pivotal for the exercise of leadership in organizations. Specifically, prudence would help in decision-making (Havard [23]; Riggio et al. [19]) by implying three behaviors: advise (search for evidence from different sources), judgment (weighting of the available evidence) and resolution (making well-balanced decisions). On the other hand, temperance would allow personal self-regulation by moderating bodily reactions in positive and negative contingencies. In this line, fortitude would allow persistence and staying calm in adversity (dangerous situations or that generate negative emotions). Finally, justice (giving each person accordingly) would allow for the development of interpersonal relationships in work contexts by favoring the acceptance of one’s own and other people’s limitations (virtue of humility), saying things as they are (truth) openly and with affection for the other (affability) and without pursuing benefits or personal gains (liberality) from the virtue of civility.

From a theoretical perspective, Riggio et al. [19] indicate that virtue-based leadership would be consistent with transformational (and its dimensions motivational inspiration, idealized influence, individual considerations and intellectual stimulation), authentic (awareness, transparent relationships, internalized morality, and balanced processing) and transactional (contingent reward, active management by exception, passive management by exception) leadership models. In turn, these authors state that virtue-based leadership would be associated with high levels of motivation, achievement and morality. Regarding this last aspect, most of the above-mentioned models have exhibit the weakness of being based on the theory of social learning, i.e., the observation of certain types of models to develop ethical leadership would be relativism-based and conflicting with any inconsistency between the values and ends that are to be achieved.

Likewise, Riggio et al. [19] report that the results of the LVQ test specify four discrete factors (one for each virtue), through two studies conducted with 300 managers from different organizations. Between the two applications, the global reliability of this instrument after applying the internal consistency method is 0.965. Similarly, the studies only reported 52% and 66.29% of explained variance, for the first and second factors, respectively. Reliability (justice, fortitude, prudence and temperance) and confirmatory factor analyses are also reported in the Method section below.

In turn, for the analysis of the validity of the test using external criteria, the authors report significant correlations with the authentic leadership tests (r = 0.90, p = 0.01. Authentic Leadership Scale, ALS. Walumbwa in Riggio et al. [19]), ethical leadership tests (r = 0.93, p = 0.01. Ethical Leadership Scale, ELS. Brown et al. in Riggio et al. [19]), transformational leadership tests (r = 0.85, p = 0.01. MultiFactor Leadership Questionnaire, MLQ-5X. Bass et al. [34]) and the Contingent Reward scale (r = 0.82, p = 0.01. MLQ).

Additionally, the authors report that the LVQ test shows negative correlation indices with *laissez-faire* leadership (r = -0.66, p = 0.01), active management by exception (r = -0.29, p = 0.01), passive management by exception (r = -0.51, p = 0.01), and narcissistic leadership (r = -0.67, p = 0.01). In turn, it is noteworthy that virtuous leadership is not related to gender leadership (r = 0.30, p>0.05), leadership level (r = 0.15, p>0.05), number of years in the current position (r = 0.14, p>0.05) and direct reports (r = -0.05, p>0.05).

In the applied context, based on the interconnection between theory, experience and research, Cardona et al. [35] and Cardona et al. [14] have proposed the development of a leadership model based on 25 competencies (grouped into business, interpersonal and personal dimensions), while Cardona et al. [5] have proposed the mission-based management model and Vilallonga [4], the leadership model of the three needs, three realities and three roles, which have been extended to the field of public organizations by Núñez [36].

From the people management approach, Cardona et al. [35] point out that different contingencies would have led organizations to develop management models oriented towards tasks (hierarchical company), objectives (professional company) and competencies (competent company). For these authors, competency-based management would be more flexible by rescuing the positive elements of the previous two types, evaluating and diagnosing competencies (IESE TD360° test), proposing an improvement plan calculated with a formula based on three criteria (importance of competency, aptitude and attitude) and developing a model of 25 competencies. IESE competencies are subdivided into business (business and organization vision, customer orientation, resource management, negotiation, networking), interpersonal (communication, conflict management, charisma, delegation, coaching, teamwork) and personal dimensions, which are, in turn, subdivided into external proactivity (creative initiative, optimism, ambition), external personal management (management of time, information, stress, self-criticism, self-knowledge, learning capacity) and internal self-government (decision-making, decisions, self-control, emotional balance and integrity).

It should be noted that from two studies referred to by Cardona et al. [35] in 2001 (multinational companies that hire MBA students, replicated in 2008) and 2007 (students from the Business School of the University of Navarra), the ten most relevant competencies (IESE TD360° test) for the management function were capacity for change, creative initiative, decision-making (prudence), communication, empowerment, customer orientation, integrity (fairness), teamwork, resilience (strength), time, and conflict management. According to the definitions proposed by the authors, a clear theoretical consistency is observed with the virtues proposed by Riggio et al. [19] and the ethical competencies put forward by Regojo [2]. However, since the psychometric data of the IESE TD360° test are not available, a comparative analysis was not possible.

Based on the literature reviewed and given the need to measure the impact that virtues have on the decision-making process of people who exercise leadership functions, this work aims to adapt the LVQ test (Riggio et al. [19]) to the Spanish language for work and organizational psychologists and people who exercise leadership functions (for evaluation by third parties of the virtues of prudence, justice, strength and temperance) and evaluate the metric properties of an instrument that can be used in the initial diagnosis and follow-up of different organizational improvement programs.

## Materials and methods

### Participants

The participants of the study (work and organizational psychologists and people who exercise leadership functions) were selected through non-probabilistic sampling, a sample by arrangement (n = 759) during the COVID-19 pandemic in Chile, with a selection error of 0.0356. and a high adequacy index for sample size (6 = 0.93).

Recruitment was carried out through job platforms (stage 1: 44.1%) and databases from different universities (stage 2: 42.3%;) and public, private and social institutions (stage 3: 13.6%) that agreed to distribute the call. Once the inclusion criteria were met, a private invitation was sent to each selected person. In the case of university institutions and other organizations that accepted to participate, the invitation was extended to recruit people who met the criteria. The final sample was sociodemographically distributed as follows: sex (men 23.3%; women 22.1%; unregistered 54.5%), years of work experience (0–2 years 16.2%; 2–5 years 9.6%; 5–10 years 13 .7%; more than 10 years 60.5%), type of organization in which they work (public 25%; private 63.8%, social 5.0%; other 6.2%). When the virtues were analyzed separately according to these variables, no statistically significant differences were observed between them.

Data collection was conducted in accordance with the guidelines of the International Test Commission (ITC) and the NORME UNE ISO 10667 [37] (in force during the course of the study), the tests were applied online (from May 01, 2021, to September 27, 2022) through a private link sent to each participant.

The research was conducted following the principles embodied in the Declaration of Helsinki and local statutory requirements Law 20.120 regarding Scientific Research in Human Beings, their Genome, and the Prohibition of Human Cloning. The Institutional Research Ethics Committee “Scientific Ethics Committee of the Vice-rectory for Research, Innovation and Creation of the University of Santiago of Chile, area of Social and Human Sciences” approved the study “Evaluation of an Explanatory Model of Workplace Inclusion of People in a Situation of Disabilities Based on Attitudes, Ethical Leadership and Psychological Well-Being (Ethics report number 071/2020, Santiago, March 26, 2020 The personal handling of data was protected and a written informed consent was used (approved by the Institutional Ethics Committee).

In addition, once responses were received, participants were thanked and feedback on the results was sent to the people who requested it. Finally, no dropouts or loss of data were recorded during the study; therefore, no data imputation procedure was necessary to avoid adverse effects in the estimation of statistical indices (Livacic-Rojas et al. [38]).

### Instruments

In the original study (explanatory design with latent variables), the LVQ test (Riggio et al. [19]) was used, which contains 19 items (second version) Likert-type items ☯from 1 (strongly disagree) to 5 (strongly agree)] that measure the virtues of prudence, temperance, justice and strength in 140 managers from different organizations. In its final version (S1 Appendix Spanish Version of Leadership Virtues), this instrument presented a global reliability through the internal consistency method of 0.97. In turn, the reliability for the dimensions of justice, strength, prudence and temperance were 0.94, 0.94, 0.92, and 0.92 respectively. Similarly, for factorial validity, 66.29% of explained variance (for the first factor) was reported with a high factorial load (0.5) of all the items, together with the comparative fit (CFI = 0.96; TFI = 0.95), parsimonious fit (RMSEA = 0.07; SRMR = 0.02), and absolute fit (χ^2^ = 2,687.62; p <0.01) indexes.

### Procedure

Through an explanatory design with latent variables (Ato et al. [39]), in a first stage, the inter-judge reliability of the LVQ test was analyzed from the translation and back-translation process following the guidelines of Muñiz et al. [40]^.^ We worked with two native Spanish-speaking translators (from Spain and Chile, respectively) and one native English-speaking translator (from England), while the two inter-judges were experts in leadership. After the language adaptation of the 19 items of the test, the two inter-judges evaluated the content based on the criteria of General Guidelines (five items), Item Format (four items), Grammar and Writing (five items), Passages (three items) and Culture (five items). (Muñiz et al. [40])^.^ According to the results obtained, agreement was statistically significant for all the items (Kappa index and Mc Nemar test, shown in the results section) with an agreement greater than 90% (Bailey et al. [41]).

Prior to the empirical collection of data, the pilot study (non-probabilistic sampling with an accidental sample with n<30 people) was conducted to facilitate the induction of future people evaluated with the test and its application to the target population. Then, data collection was conducted in the three stages above mentioned through job platforms (stage 1, n = 321); databases from different universities (stage 2, n = 335) and in public, private and social institutions (stage 3, n = 103).

### Data analysis

The data were analyzed with the statistical software SAS 9.4 [42]. In a first stage, the discrimination and location indices of the items were analyzed, as well as the reliability and validity of the item scores (analysis of the homogeneity and communality of the items) and graphs (heat maps, characteristic curves) and item information. In a second stage, for the inferential analysis of the item scores with the IRT, the polychoric correlation indices were used (contrast of hypotheses with the Wald Likelihood test) as well as graphic techniques, characteristic and informative curves of the items (O’Rourke et al. [43]). In a third stage, the dimensions of the test reliability (Cronbach’s Alpha and Tucker Lewis statistical techniques), validity (statistical techniques, quadratic canonical correlation coefficients, and factor analysis for consistency between theoretical and empirical factor structures; graphic techniques: sediment graphs) and the mean square error for the diagonal of the residual matrix were evaluated. In the fourth stage, the fit of the first- and second-order model was evaluated through five sub-stages, namely specification, identification, estimation, evaluation, and re-specification of the model. In this analytical context, for the adjustment of the model, four evaluation criteria associated with the respective theoretical ranges were followed for the inferential decision and subsequent discussion ☯(see criteria proposed by Pérez [44], O’Rourke et al. [43] and Abad et al. [45])]: a) Analysis of the absolute statistical adjustment ☯χ^2^, υ (degrees of freedom) p>0.05)]; b) Analysis of the comparative statistical fit: AGFI ☯with theoretical ranges as follows; AGFI≥0.95 or more (optimal); 0.90≤AGFI≤0.94 (acceptable); AGFI<0.90 (poor)]; CFI ☯Comparative Fit Index where CFI>0.95 or more (optimal); 0.90< CFI<0.94 (acceptable); CFI<0.90 (poor)]; TLI ☯Tucker Lewis Index, where, TLI>0.95 or more (optimal); 0.90<TLI<0.94, (acceptable); TLI<0.90 (poor)]; c) Analysis of the parsimonious statistical fit: ☯SRMR, Normalized root of the residual mean square with theoretical range of 0.05<SRMR< 0.079 (acceptable); 0.08< SRMR <0.099 (marginally acceptable); SRMR>0.10 (poor)]; RMSEA ☯Root mean square error of approximation with RMSEA. Theoretical range 0.06–0.08, where RMSEA<0.06 (optimal); 0.061<RMSEA<0.080; RMSEA>0.081 (poor)]; d) Cross Validity Index Estimation: ECVI [Expected Cross-Validation Index (0.01≤CVI≤0.99] and Graphic Techniques: Path diagram.

The database of this study is available at the following link https://doi.org/10.7910/DVN/AEX8PP

## Results

Regarding the expert judge analysis for the consistency of item content (evaluation of 381 responses), in the translation stage, the Kappa (IK = 0.65, SE = 0.07; IC = 0.51;0.79), and the statistical significance indexes were calculated through the Mc Nemar test (χ^2^(1) = 21.00, *p<0*.*0001*). In turn, in the back-translation stage, the Kappa and the statistical significance indexes were (IK = 0.42, SE = 0.05; IC = 0.33;0.52) and (χ^2^(1) = 78.00, *p<0*.*0001*) respectively.

Based on the results (Table 1), 89.75% (17 items out of a total of 19) present average scores of four (on a response scale between one and five) and a range of response variation of 0.57 (between the highest and lowest deviations). According to the theoretical ranges of Lind et al. [46], in general, the items show a tendency towards high scores. In specific terms, in relation to the slight bias of the items (±0.00;±1.00), 15.79% (three items) are negative and 5.26% are positive. Similarly, moderate bias (±1.01;±2.00) is negative in 52.63% (10 items) and positive in only 5.26% (1 item). Regarding high bias (±2.01;±3.00), 15.78% (three items) is negative, while 5.26% (one item) presents a very high negative bias (±3.01;±4.00).

**Table 1:** Statistical descriptive and item response theory indexes of the items of the leadership test based on virtues in a sample of work and organizational psychologists and people who exercise leadership functions.

IT	AA	SD	σ ^3^	σ^4^	ID	SE	p	LI	SE	p	D	L
1	4.39	0.73	1.39	3.04	0.94	0.07	0.00	0.04	0.06	0.00	ACP	PMA
2	4.15	1.23	-1.4	0.71	0.76	0.06	0.00	-0.28	0.07	0.00	ACP	PMB
3	4.22	1.00	-1.3	1.02	0.60	0.05	0.00	-1.63	0.16	0.00	MAC	PMB
4	4.24	1.14	-1.5	1.16	0.76	0.06	0.00	-0.38	0.08	0.01	ACP	PMB
5	4.21	0.94	-1.4	1.74	0.89	0.07	0.00	0.15	0.07	0.00	ACP	PMA
6	3.74	1.24	-0.8	-0.3	0.60	0.05	0.00	-0.76	0.11	0.00	MAC	PMB
7	4.09	1.10	-1.1	0.13	0.38	0.05	0.00	-1.89	0.26	0.01	INC	PMB
8	4.32	0.98	-1.5	1.59	0.89	0.07	0.00	-0.29	0.07	0.00	ACP	PMB
9	4.20	1.11	-1.4	1.04	0.70	0.06	0.00	-0.19	0.07	0.00	ACP	PMB
10	4.12	0.95	-0.9	0.39	0.52	0.05	0.00	0.36	0.10	0.00	INC	PMB
11	4.17	1.11	-1.3	0.72	0.67	0.06	0.00	-0.19	0.08	0.00	ACP	PMB
12	2.48	1.23	0.35	-0.9	0.06	0.04	0.07	3.00	0.41	0.78	INC	PMA
13	2.98	1.27	-0.0	-1.1	-0.03	0.07	0.24	10.6	14.9	0.24	INC	PMB
14	4.53	0.86	-2.2	4.83	0.96	0.08	0.00	-0.69	0.08	0.00	ACP	PMB
15	4.69	0.76	-3.1	10.1	1.37	0.12	0.00	-0.88	0.12	0.00	ACP	PMB
16	4.46	1.06	-2.1	3.60	0.63	0.06	0.00	-1.13	0.13	0.00	ACP	PMB
17	4.68	0.79	-3.0	8.98	1.24	0.11	0.00	-1.08	0.11	0.00	ACP	PMB
18	4.36	0.99	-1.8	2.76	0.75	0.06	0.00	-0.44	0.08	0.00	ACP	PMB
19	4.29	1.30	-1.7	1.39	0.65	0.06	0.00	-1.00	0.11	0.00	ACP	PMB

IT (Item); AA (Arithmetic Average); SD (Standard Deviation); σ^3^ (Skewness); σ^4^ (Kurtosis); ID (Item Discrimination Index); LI (Location Index); SE (Standard Error); p (Exact Probability); D (Decision); L (Location); ACP (Acceptable); MAC (Marginally Acceptable); INC (Unacceptable); PMB (Low Moderate Score, –2.0; -0.01); PB (Low score -4;-2.01); PMA (Moderate High Score, +0.01; +2.00); PA (High Score +2.00; +4.00).

With respect to the pointing of the data (Table 1), according to the theoretical ranges of Lind et al. [46], the items show in general a moderate concentration that is slightly above the normal curve. In specific terms, the slight pointing of the items (±0.00;±1.00) shows that 21.05% (four items) is positive and 15.78% is negative. In the same way, regarding moderate pointing (±1.01;±2.00), 31.58% (6 items) is positive and only 5.26% (1 item) is negative. Regarding high targeting (±2.01;±3.00), 5.26% (one item) is positive, while 10.53% (two items) present a very high negative bias (±3.01;±4.00), and 15.79% (three items) presents a severe pointing (-4.00<σ^4^<+4.00).

In accordance with the above and as described in the following paragraphs (Table 1), it should be noted that the bias and pointing indices of the items belong mainly to the justice dimension, and their statistical indices place them within the acceptable ranges.

In the case of item parameters (Table 1), 68.42% (13 items out of 19 of the total test) have acceptable parameters as they are within the theoretical ranges of discrimination, location index, and error standard below 0.30, with statistically significant probability values of the hypothesis test. Of these 13 items, three items correspond to the "prudence" dimension, three to the "fortitude" dimension, one to the "temperance" dimension, and five to the "justice" dimension. In turn, 10.53% (two items) would have marginally acceptable parameters as they are within the peripheral limits of the discrimination ranges, location index and standard error below 0.30 (in two out of four) with statistically significant probability values. Finally, (Table 1), 21.05% (four items) have unacceptable parameters as they are below the theoretical ranges of discrimination index and standard error below 0.30, with statistically significant probability values. Of this subgroup, one item corresponds to the "fortitude" dimension, two to "fortitude" and "temperance", respectively, and one to the "justice" dimension.

In turn (Table 2), the polychoric correlations between the test items are statistically significant in 137 (83.03%) of the 165 conditions analyzed. In this context, of the total of 19 items, only items 12 and 13 show indices with no statistical significance. In this sense, under these conditions, the remaining 17 items would point in the same direction to measure the Ethical Leadership construct based on Virtues.

**Table 2:** Statistical indexes items of polychoric correlations of the leadership test based on virtues in a sample of work and organizational psychologists and people who exercise leadership functions.

It	r	WT			LR		Items	r	WT			LR	
		SE	χ^2^	p	χ^2^	p			SE	χ^2^	p	χ^2^	p
1–2	0.33	0.04	58.27	0.00	50.22	0.00	2–18	0.36	0.04	66.08	0.00	55.50	0.00
1–3	0.30	0.04	46.03	0.00	41.30	0.00	2–19	0.45	0.04	108.5	0.00	81.85	0.00
1–4	0.41	0.04	99.78	0.00	80.08	0.00	3–4	0.52	0.04	208.9	0.00	141.7	0.00
1–5	0.56	0.03	264.7	0.00	166.4	0.00	3–5	0.34	0.04	69.04	0.00	58.83	0.00
1–6	0.34	0.04	71.69	0.00	60.59	0.00	3–6	0.14	0.04	10.34	0.00	10.01	0.00
1–7	0.23	0.04	25.81	0.00	23.90	0.00	3–7	0.19	0.04	18.16	0.00	17.38	0.01
1–8	0.46	0.04	133.2	0.00	100.1	0.00	3–8	0.44	0.04	127.0	0.00	96.50	0.00
1–9	0.39	0.04	86.65	0.00	71.47	0.00	3–9	0.36	0.04	75.15	0.00	62.70	0.00
1–10	0.30	0.04	48.61	0.00	43.11	0.00	3–10	0.28	0.04	43.14	0.00	38.67	0.00
1–11	0.41	0.04	104.6	0.00	83.74	0.00	3–11	0.35	0.04	69.82	0.00	59.46	0.00
1–12	0.05	0.04	1.38	0.24	1.33	0.25	3–12	-0.06	0.04	2.07	0.15	2.00	0.16
1–13	0.01	0.04	0.03	0.86	0.03	0.87	3–13	-0.08	0.04	3.99	0.05	3.86	0.05
1–14	0.53	0.04	179.5	0.00	121.7	0.00	3–14	0.24	0.05	25.97	0.00	24.42	0.00
1–15	0.65	0.04	314.2	0.00	163.4	0.00	3–15	0.40	0.05	69.07	0.00	55.57	0.00
1–16	0.30	0.05	68.13	0.00	58.17	0.00	3–16	0.34	0.05	53.45	0.00	46.83	0.00
1–17	0.63	0.04	264.6	0.00	150.4	0.00	3–17	0.42	0.05	75.85	0.00	60.03	0.00
1–18	0.51	0.04	176.3	0.00	122.9	0.00	3–18	0.23	0.05	25.69	0.00	23.92	0.00
1–19	0.37	0.05	64.62	0.00	55.35	0.00	3–19	0.39	0.04	77.00	0.00	63.61	0.00
2–3	0.51	0.04	200.1	0.00	136.9	0.00	4–5	0.44	0.04	126.3	0.00	96.81	0.00
2–4	0.59	0.03	309.3	0.00	178.5	0.00	4–6	0.25	0.04	33.89	0.00	31.04	0.00
2–5	0.42	0.04	110.8	0.00	86.10	0.00	4–7	0.21	0.05	20.83	0.00	19.53	0.00
2–6	0.27	0.04	41.22	0.00	37.12	0.00	4–8	0.44	0.04	116.4	0.00	89.37	0.00
2–7	0.27	0.04	37.23	0.00	33.83	0.00	4–9	0.39	0.04	88.09	0.00	71.56	0.00
2–8	0.50	0.04	177.2	0.00	121.3	0.00	4–10	0.29	0.04	47.02	0.00	41.89	0.00
2–9	0.33	0.04	57.71	0.00	48.94	0.00	4–11	0.33	0.04	59.35	0.00	51.44	0.00
2–10	0.32	0.04	57.57	0.00	50.04	0.00	4–12	-0.01	0.05	0.09	0.75	0.09	0.76
2–11	0.31	0.04	51.80	0.00	41.06	0.00	4–13	0.03	0.04	0.44	0.51	0.44	0.51
2–12	-0.02	0.04	0.15	0.70	0.15	0.70	4–14	0.36	0.05	60.56	0.00	51.59	0.00
2–13	0.01	0.04	0.06	0.80	0.06	0.81	4–15	0.42	0.05	76.82	0.00	59.91	0.00
2–14	0.39	0.05	76.94	0.00	62.87	0.01	4–16	0.40	0.05	75.32	0.00	61.22	0.00
2–15	0.45	0.05	91.84	0.00	69.22	0.00	4–17	0.46	0.05	97.79	0.00	72.98	0.00
2–16	0.39	0.05	71.41	0.00	57.22	0.00	4–18	0.32	0.05	51.63	0.00	45.05	0.00
2–17	0.50	0.05	126.2	0.00	87.07	0.00	4–19	0.45	0.04	107.3	0.00	81.20	0.00
5–6	0.42	0.04	132.0	0.00	100.6	0.00	7–16	0.22	0.05	19.06	0.00	17.73	0.00
5–7	0.17	0.04	15.15	0.00	14.59	0.00	7–17	0.24	0.05	20.24	0.00	18.52	0.00
5–8	0.41	0.04	104.2	0.00	81.78	0.00	7–18	0.24	0.04	27.44	0.00	24.94	0.00
5–9	0.32	0.04	56.24	0.00	48.39	0.00	7–19	0.23	0.05	21.87	0.00	20.39	0.00
5–10	0.33	0.04	68.25	0.00	57.62	0.00	8–9	0.51	0.04	197.2	0.00	132.9	0.00
5–11	0.34	0.04	66.14	0.00	56.94	0.00	8–10	0.42	0.04	117.9	0.00	90.68	0.00
5–12	-0.01	0.04	0.02	0.89	0.02	0.90	8–11	0.40	0.04	96.67	0.00	78.02	0.00
5–13	0.08	0.04	3.86	0.05	3.75	0.05	8–12	0.02	0.04	0.22	0.64	0.21	0.65
5–14	0.54	0.04	210.8	0.00	135.7	0.00	8–13	-0.10	0.04	5.25	0.02	5.09	0.02
5–15	0.60	0.04	236.7	0.00	137.9	0.00	8–14	0.47	0.04	127.8	0.00	94.07	0.00
5–16	0.36	0.05	60.16	0.00	51.34	0.00	8–15	0.52	0.04	137.9	0.00	94.79	0.00
5–17	0.59	0.04	223.9	0.00	134.0	0.00	8–16	0.37	0.05	60.95	0.00	50.83	0.00
5–18	0.45	0.04	132.8	0.00	99.57	0.00	8–17	0.53	0.04	150.9	0.00	100.7	0.00
5–19	0.32	0.05	45.52	0.00	40.34	0.00	8–18	0.39	0.04	84.03	0.00	68.21	0.00
6–7	0.10	0.04	4.99	0.03	4.84	0.03	8–19	0.46	0.04	114.6	0.00	85.42	0.00
6–8	0.30	0.04	52.85	0.00	46.03	0.00	9–10	0.34	0.04	68.04	0.00	57.72	0.00
6–9	0.31	0.04	59.74	0.00	50.46	0.00	9–11	0.41	0.04	108.2	0.00	85.79	0.00
6–10	0.24	0.04	34.34	0.00	31.06	0.00	9–12	-0.04	0.04	1.06	0.30	1.01	0.32
6–11	0.32	0.04	64.56	0.00	55.09	0.00	9–13	-0.07	0.04	2.41	0.12	2.36	0.12
6–12	0.22	0.04	30.78	0.00	27.55	0.00	9–14	0.35	0.05	59.91	0.00	50.79	0.00
6–13	-0.01	0.04	0.12	0.73	0.12	0.73	9–15	0.50	0.04	123.7	0.00	88.36	0.00
6–14	0.40	0.04	95.78	0.00	76.80	0.00	9–16	0.39	0.05	73.01	0.00	59.40	0.00
6–15	0.55	0.04	191.4	0.00	120.4	0.00	9–17	0.52	0.04	138.5	0.00	94.79	0.00
6–16	0.32	0.04	49.60	0.00	43.27	0.00	9–18	0.33	0.04	57.99	0.00	50.02	0.00
6–17	0.47	0.04	113.7	0.00	84.06	0.00	9–19	0.37	0.05	64.17	0.00	53.59	0.00
6–18	0.40	0.04	99.82	0.00	80.73	0.00	10–11	0.29	0.04	48.23	0.00	42.90	0.00
6–19	0.24	0.04	26.22	0.00	24.63	0.00	10–12	0.00	0.04	0.00	0.94	0.00	0.95
7–8	0.43	0.04	124.1	0.00	95.48	0.00	10–13	0.00	0.04	0.00	0.97	0.00	0.97
7–9	0.29	0.04	47.87	0.00	42.84	0.00	10–14	0.26	0.05	30.70	0.00	27.70	0.00
7–10	0.32	0.04	64.62	0.00	55.72	0.00	10–15	0.32	0.05	40.80	0.00	35.79	0.00
7–11	0.24	0.04	31.20	0.00	28.51	0.00	10–16	0.33	0.05	51.20	0.00	45.01	0.00
7–12	-0.04	0.04	1.12	0.29	1.08	0.30	10–17	0.31	0.05	37.06	0.00	32.76	0.00
7–13	-0.02	0.04	0.31	0.58	0.30	0.58	10–18	0.27	0.05	36.71	0.00	33.23	0.00
7–14	0.28	0.05	37.52	0.00	33.43	0.00	10–19	0.33	0.05	52.22	0.00	45.77	0.00
7–15	0.19	0.05	12.93	0.00	12.19	0.00	11–12	0.17	0.04	16.05	0.00	14.73	0.00
11–13	-0.18	0.04	18.37	0.00	17.13	0.00	13–18	0.02	0.04	0.30	0.58	0.30	0.58
11–14	0.42	0.04	95.78	0.00	27.70	0.00	13–19	-0.08	0.05	2.77	0.10	2.77	0.10
11–15	0.51	0.04	133.1	0.00	93.54	0.00	14–15	0.67	0.04	355.9	0.00	166.7	0.00
11–16	0.40	0.05	78.27	0.00	64.00	0.00	14–16	0.48	0.04	110.6	0.00	81.64	0.00
11–17	0.50	0.04	124.4	0.00	90.77	0.00	14–17	0.64	0.04	279.3	0.00	148.4	0.00
11–18	0.34	0.04	61.38	0.00	53.54	0.00	14–18	0.57	0.04	229.9	0.00	143.3	0.00
11–19	0.34	0.05	54.05	0.00	46.83	0.00	14–19	0.43	0.05	83.97	0.00	67.34	0.00
12–13	-0.05	0.04	1.85	0.17	1.80	0.18	15–16	0.55	0.05	141.9	0.00	93.73	0.00
12–14	0.03	0.05	0.35	0.56	0.33	0.56	15–17	0.83	0.02	1208	0.00	282.5	0.00
12–15	0.03	0.05	0.42	0.52	0.41	0.52	15–18	0.57	0.04	195.6	0.00	119.8	0.00
12–16	0.03	0.05	0.36	0.55	0.35	0.56	15–19	0.52	0.05	124.1	0.00	86.00	0.00
12–17	-0.02	0.05	0.15	0.70	0.14	0.70	16–17	0.54	0.05	134.8	0.00	90.49	0.00
12–18	0.16	0.04	13.74	0.00	13.18	0.00	16–18	0.37	0.05	62.83	0.00	52.59	0.00
12–19	0.04	0.05	0.59	0.44	0.57	0.45	16–19	0.48	0.05	110.8	0.00	80.98	0.00
13–14	-0.03	0.05	0.30	0.59	0.29	0.59	17–18	0.54	0.04	157.1	0.00	103.5	0.00
13–15	-0.03	0.05	0.43	0.51	0.42	0.52	17–19	0.53	0.05	125.0	0.00	85.36	0.00
13–16	-0.15	0.05	10.56	0.00	10.15	0.00	18–19	0.34	0.05	50.14	0.00	43.55	0.00
13–17	-0.06	0.05	1.20	0.28	1.22	0.27							

IT (Item); WT (Wald Test); r (Polychoric Correlation); SE (Standard Error); χ^2^ (Observed Chi Square); p (Exact Probability); LR (Likelihood Ratio Test).

In line with the above (Table 3), the correlations between the dimensions of the test are low (r <0.66), which would indicate that in the presence of an explained variance of less than 0.4356, it would be possible to conjecture that they are independent of each other.

**Table 3:** Statistical indices of correlations between the dimensions correlations of the Leadership Virtues Questionnaire in a sample of work and organizational psychologists and people who exercise leadership functions.

Dimensions	Prudence	Fortitude	Temperance	Justice
Prudence	1.00			
Fortitude	0.58	1.00		
Temperance	0.22	0.23	1.00	
Justice	0.64	0.59	0.24	1.00

**Fig 1:**
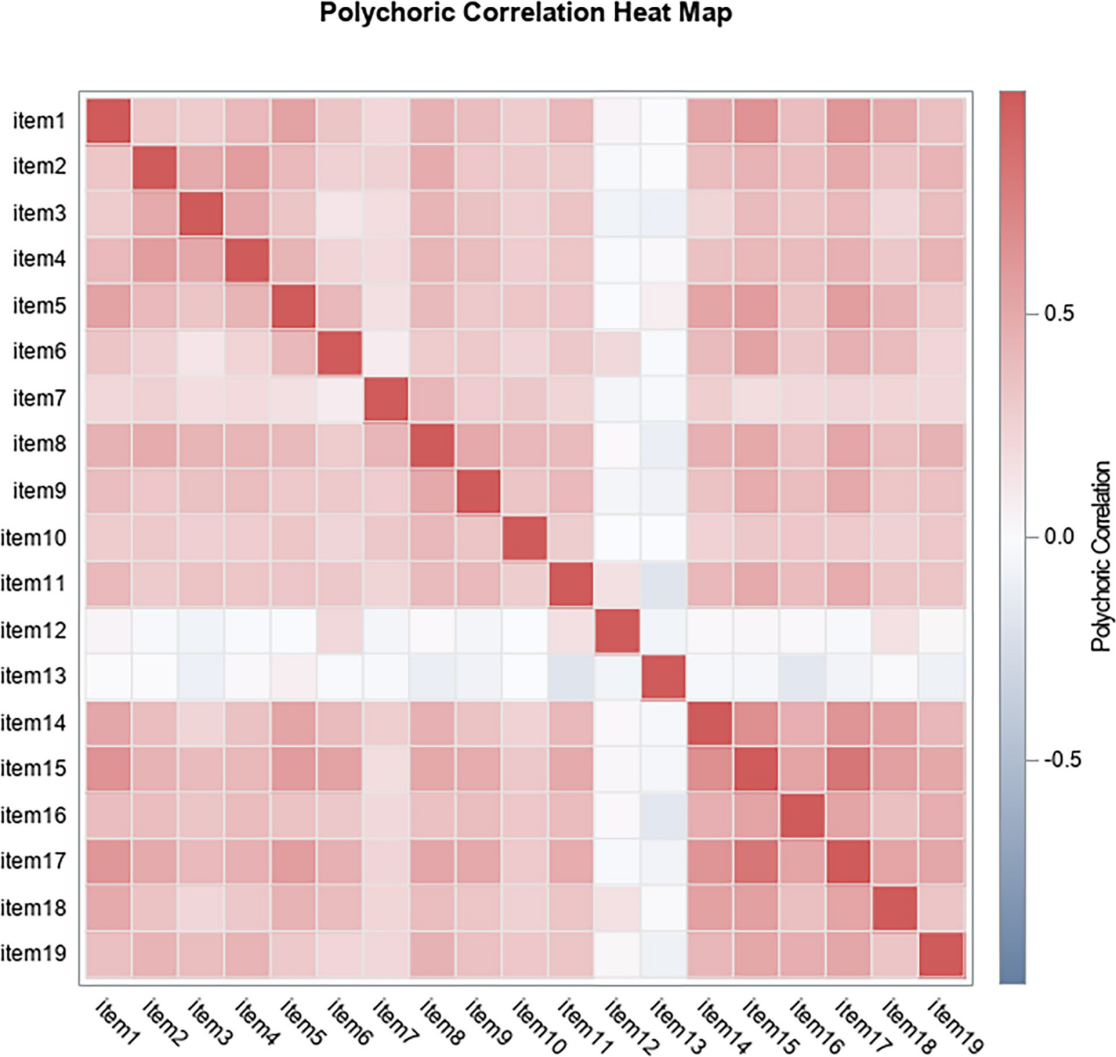
Heat map for the configuration of the Leadership Virtues Questionnaire in a sample of work and organizational psychologists and people who exercise leadership functions.

In line with the above, the heat map (Fig 1) shows that the virtue of justice presents the clearest and most defined configuration (higher correlation coefficients between them for the items 14 to 19). In turn, the virtues of prudence and strength would be located at a somewhat more diffuse level (less defined configuration). Finally, the virtue of temperance would be located at the level of least clarity (indefinite or absent configuration), which, as indicated above, is the one that shows the elements with the lowest levels of discrimination and the highest standard error for its indices.

**Fig 2:**
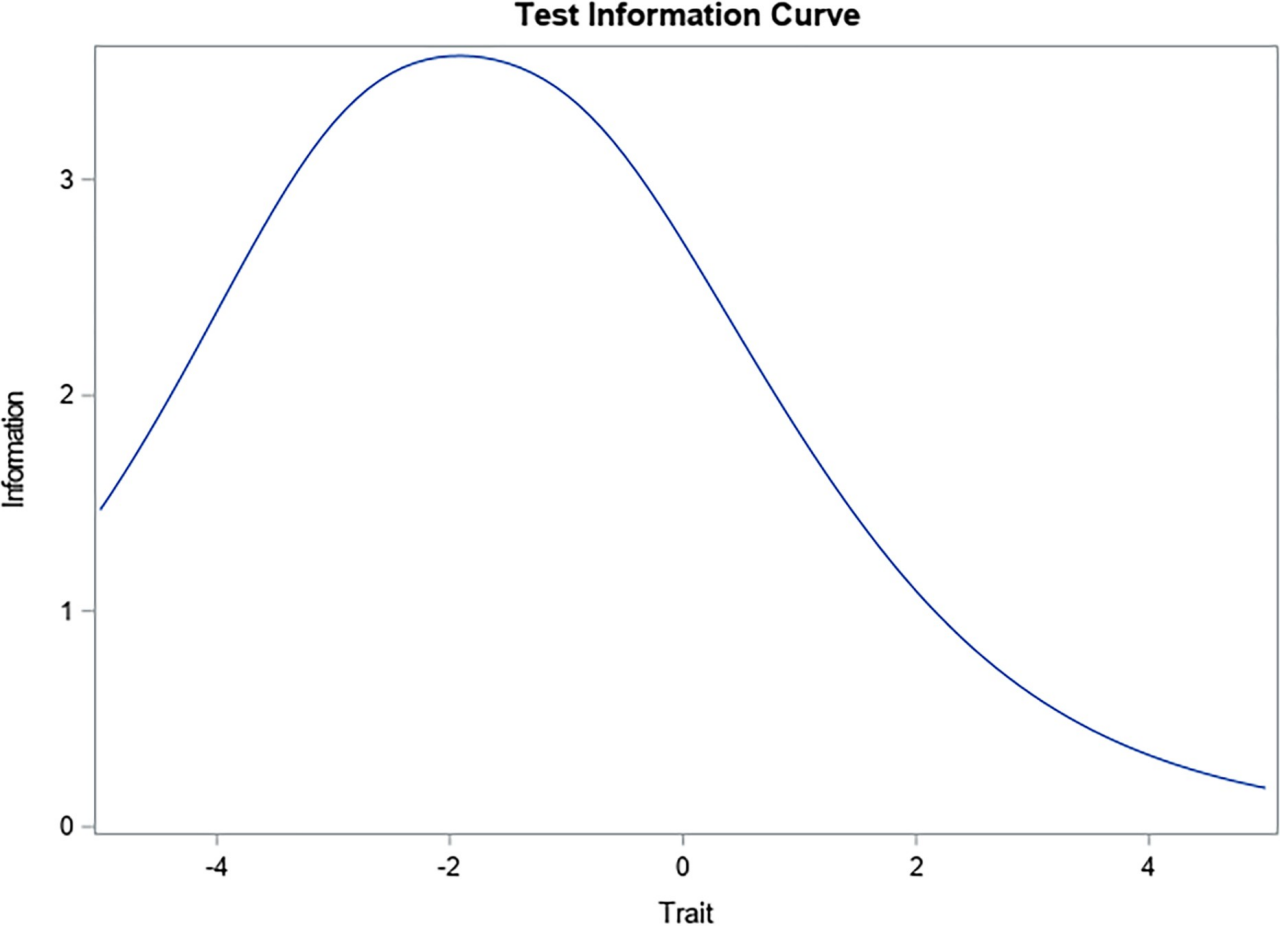
Information curve of the Leadership Virtues Questionnaire in a sample of work and organizational psychologists and people who exercise leadership functions.

Along the lines of the above, the test information curve (Fig 2) shows that the concentration of scores tends towards low moderate scores (when located from 0 to -4), which would also be associated with a greater presence of error in location indices of -4.

### Analysis of the first level fit of the model with four factors through consistency analysis between the theoretical and empirical factor structure

**Fig 3:**
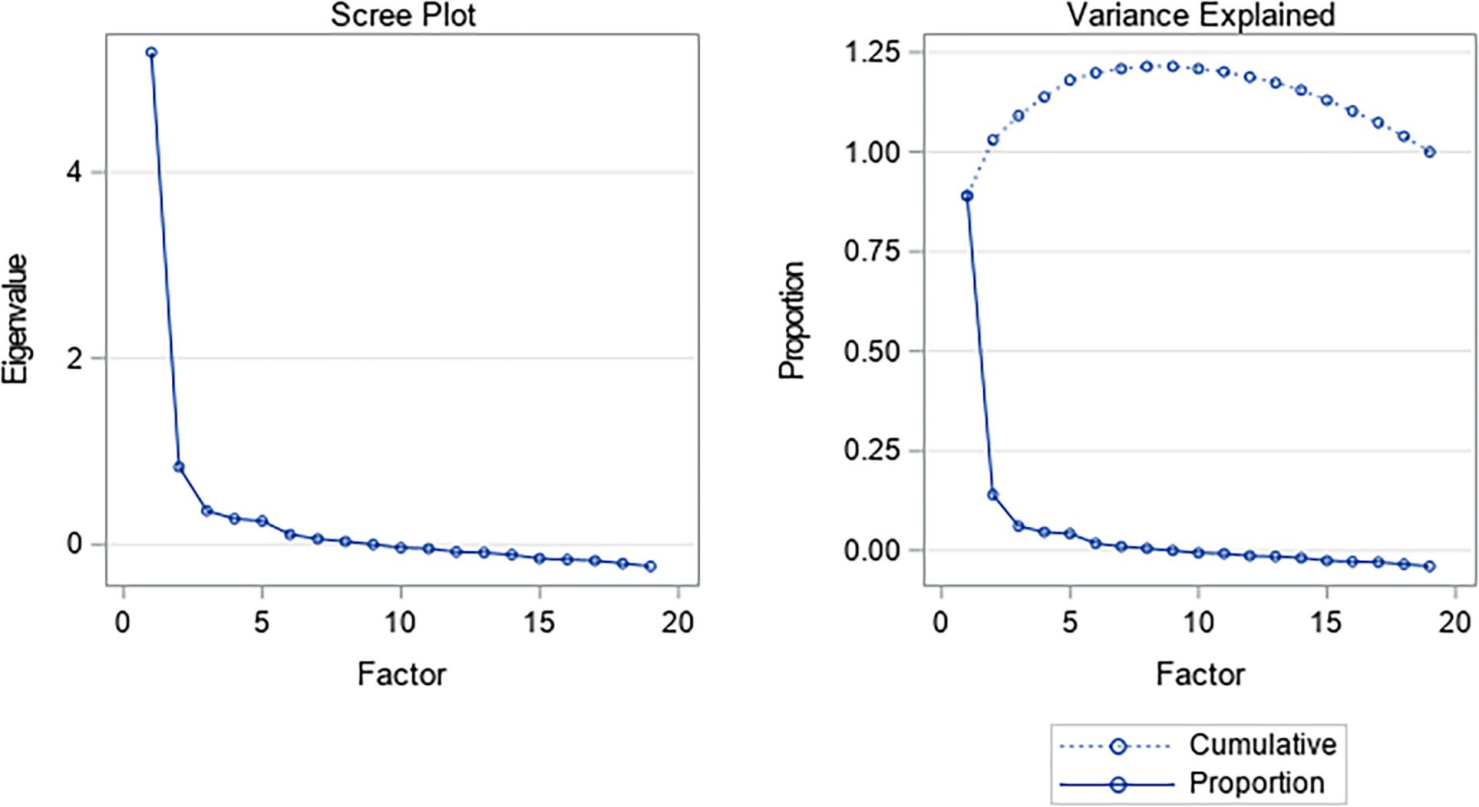
Scree plot of the Leadership Virtues Questionnaire in a sample of work and organizational psychologists and people who exercise leadership functions.

Based on the results observed for a four-dimensional hypothesis test, there would be an adequate sample size (KMO = 0.92) and consistency between the theoretical and empirical factorial structure ☯χ^2^ = 191.66 (101), *p = 0*.*01*]. In this context, according to the criteria established by O’Rourke et al. [43]., global reliability with the Tucker Lewis method is 0.98 (versus α = 0.84), while specific reliability by dimensions is: Prudence (0.72), Fortitude (0.63), Temperance (-0.05) and Justice (0.76). Additionally, the validity of the dimensions (through quadratic canonical correlations) would be: 0.92 (justice), 0.65 (prudence), 0.41 (fortitude) and 0.37 (temperance). In turn, the Scree Plot (Fig 3) shows that the final estimates of the variance would be 96.27%, with each dimension explaining 59.13, 14.96, 11.76, and 10.42, respectively.

## Analysis of model fit with four factors using first-order factor analysis

Based on the criteria proposed by Pérez [44], O’Rourke et al. [43] and Abad et al. [45], the results (Table 4) indicate that the 19-item model with the maximum likelihood estimation model shows inefficient absolute and comparative fits (statistical indices would show a significant discrepancy between the data matrix and the matrix generated in the model and located in ranges lower than 0.90), while it is efficient for the parsimonious model (0.06 according to the theoretical ranges). In turn, estimates of the cross-validity index (ECVI) slightly higher than 1.0 would affect the generalizability of virtue-based leadership indicators.

**Table 4:** Statistical indices for first-order confirmatory factor analysis to assess the fit of the model in the Leadership Virtues Questionnaire in a sample of work and organizational psychologists and people who exercise leadership functions.

Absolute Fit			Comparative Fit		Parsimonious Fit		ECVI
χ^2^	υ	p	CFI	TLI	SRMR	RMSEA	
565.02	142	0.00	0.89	0.87	0.06	0.06	0.86

χ^2^ (Chi square statistic); υ (degrees of freedom); CFI ☯Comparative Fit Index where CFI≥0.95 or more (OPTIMUM); 0.9 0≥ CFI≥0.94 (ACCEPTABLE); CFI<0.90 (POOR)]; TLI ☯Tucker Lewis Index, where, TLI≥0.95 or more (OPTIMUM); 0.90≥ TLI≥0.94, (ACCEPTABLE); TLI<0.90 (POOR)]; SRMR ☯Normalized root mean square residual with theoretical range of 0.05<SRMR< 0.079 (ACCEPTABLE); 0.08< SRMR <0.099 (MARGINALLY ACCEPTABLE); SRMR>0.10 (POOR)]; RMSEA ☯Root mean square error of approximation with RMSEA theoretical range 0.06–0.08, where RMSEA<0.06 (BEST); 0.061<RMSEA<0.080; RMSEA>0.081 (POOR)]; ECVI☯ Expected Cross-Validation Index (0.01≤CVI≤0.99; Cross Validity Index Estimate closest to ACCEPTABLE)]

**Fig 4:**
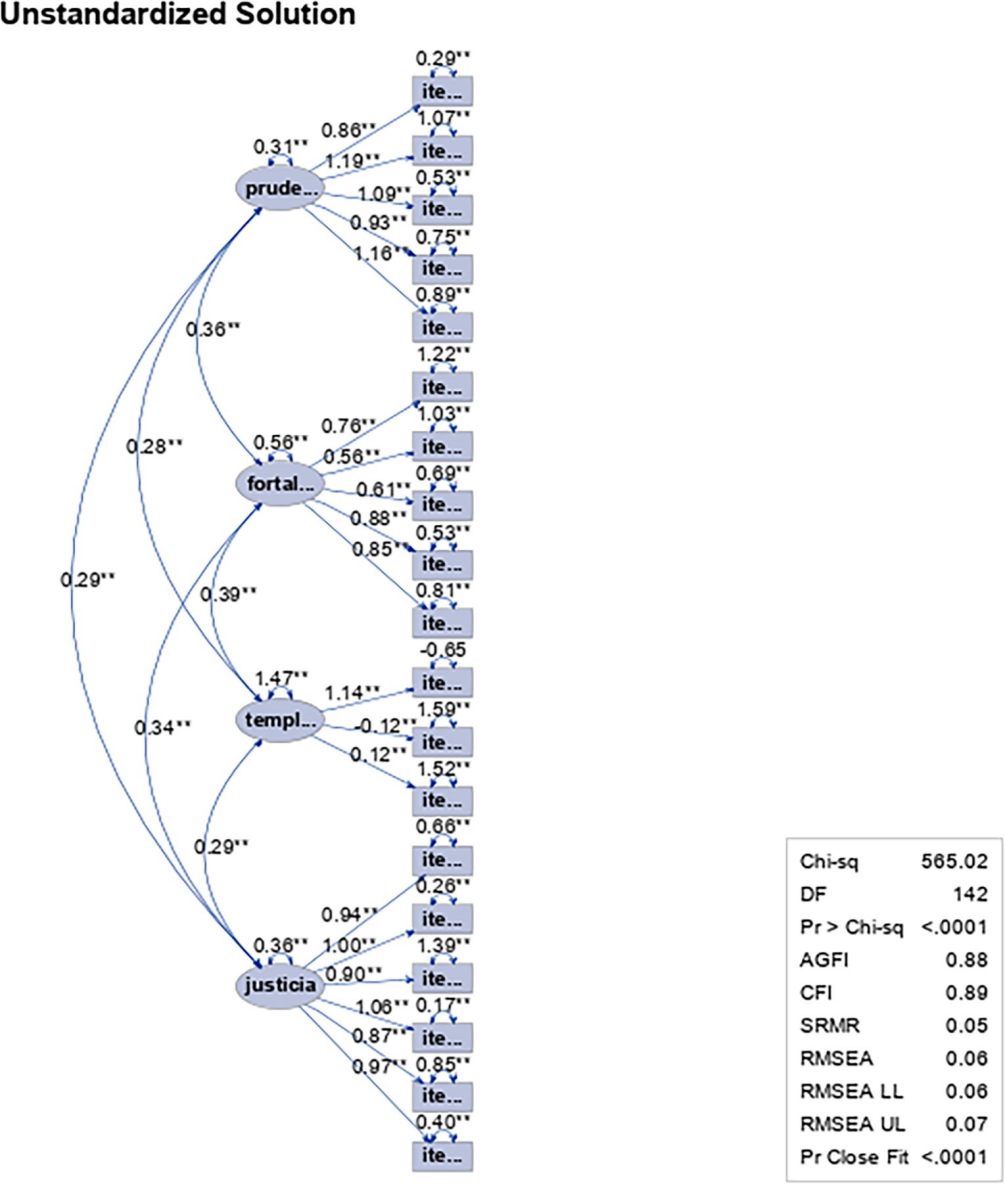
Path diagram for the first-order factorial analysis of the Leadership Virtues Questionnaire with 19 items and four dimensions in a sample of work and organizational psychologists and people who exercise leadership functions.

As a complement to the above, the inefficient adjustment of the model is exposed from the statistical indices that are reported in the path diagram (Fig 4).

**Fig 5:**
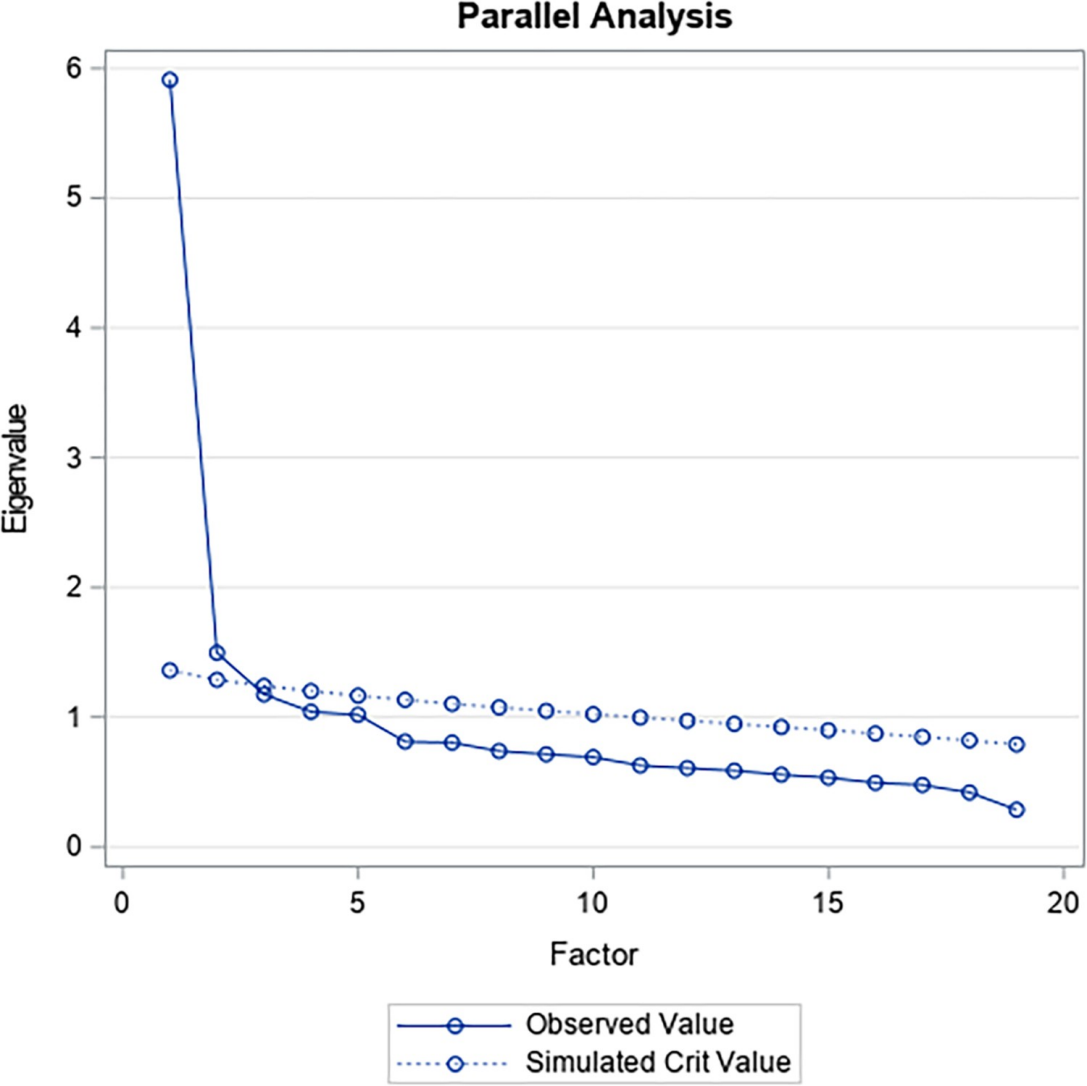
Factor retention graph through parallel analysis with the MAP4 minimum partial correlation method for scores on the Leadership Virtues Questionnaire with four dimensions in a sample of work and organizational psychologists and people who exercise leadership functions.

However, the parallel analysis carried out (Fig 5), which considers the convergence of self -evaluated values and the number of factors through 10,000 simulations, yield a (MAP4) that would explain 74.09% of the variance (p = 0.000). This analytical situation would be partially consistent with the heat map (Fig 1).

### Analysis of model fit with four factors using second-order factor analysis

According to the criteria proposed by Pérez [44], O’Rourke et al. [43] and Abad et al. [45], Table 5 shows that the global model with the 19 elements presents a marginally efficient adjustment. Specifically, the absolute adjustment exhibits a significant discrepancy, the comparative adjustment is slightly inefficient (less than 0.95) and the parsimonious adjustment has indices within optimal ranges. In turn, according to the explained variance criteria for factorial loads, 21.05% ☯items 2 (prudence), 11 (temperance), 15 and 17 (justice)] of the test elements present high factorial loads (0.75–1 of the explained variance). Similarly, 63.16% ☯items 1,3,4 and 5 (prudence), 6, 8, 9 and 10 (strength) and 14, 16, 18 and 19 (justice)] of the test elements have a moderate factorial load (0.50–0.74 of the explained variance). At a low level of explained variance (0.00–0.49), 15.79% ☯items 7 (strength) are observed; 12 and 13 (temperance)]. In addition, the factor load of the dimensions of prudence, strength and justice is higher than that corresponding to the dimension of temperance. However, the observed values of the adjustment indices should be considered with caution since the size of the estimates of the cross-validity index (ECVI) would be slightly higher than 2.0, which would affect the possibility of generalizing the leadership indicators based on the virtues.

**Table 5:** Statistical indices for second-order confirmatory factor analysis to evaluate the fit of the model in the Leadership Virtues Questionnaire in a sample of work and organizational psychologists and people who exercise leadership functions.

Absolute Fit			Comparative Fit		Parsimonious Fit		ECVI
χ^2^	υ	p	CFI	TLI	SRMR	RMSEA	
1052.0	149	0.00	0.86	0.84	0.06	0.09	1.50

See legend of Table 4.

**Fig 6:**
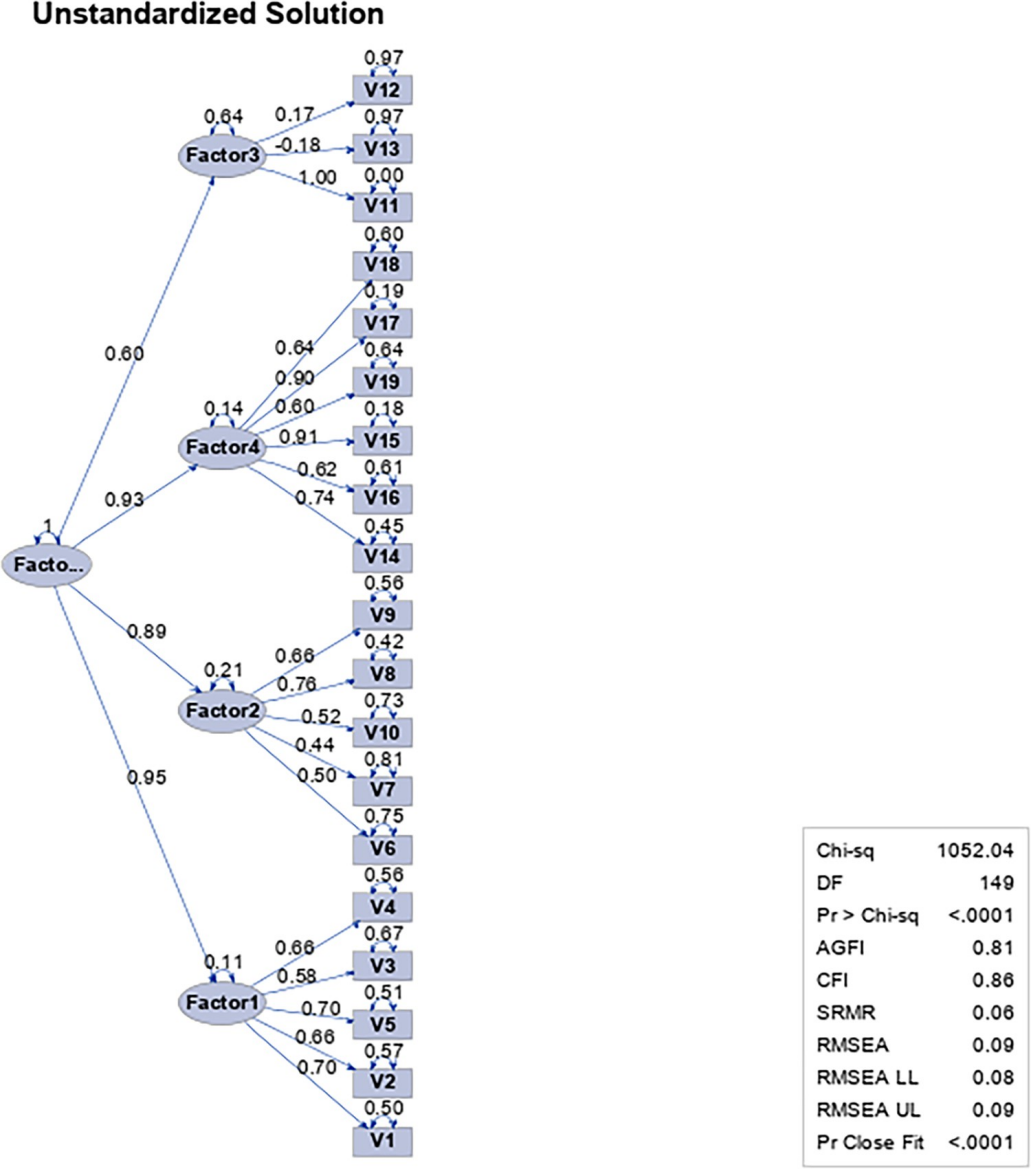
Path diagram for the second-order factorial analysis of the Leadership Virtues Questionnaire with 19 items and four dimensions in a sample of work and organizational psychologists and people who exercise leadership functions.

As a complement to the above, an efficient adjustment of the model is observed from the statistical indices in the path diagram (Fig 6).

Prudence (Factor 1); Fortitude (Factor 2); Temperance (Factor 3); Justice (Factor 4).

**Fig 7:**
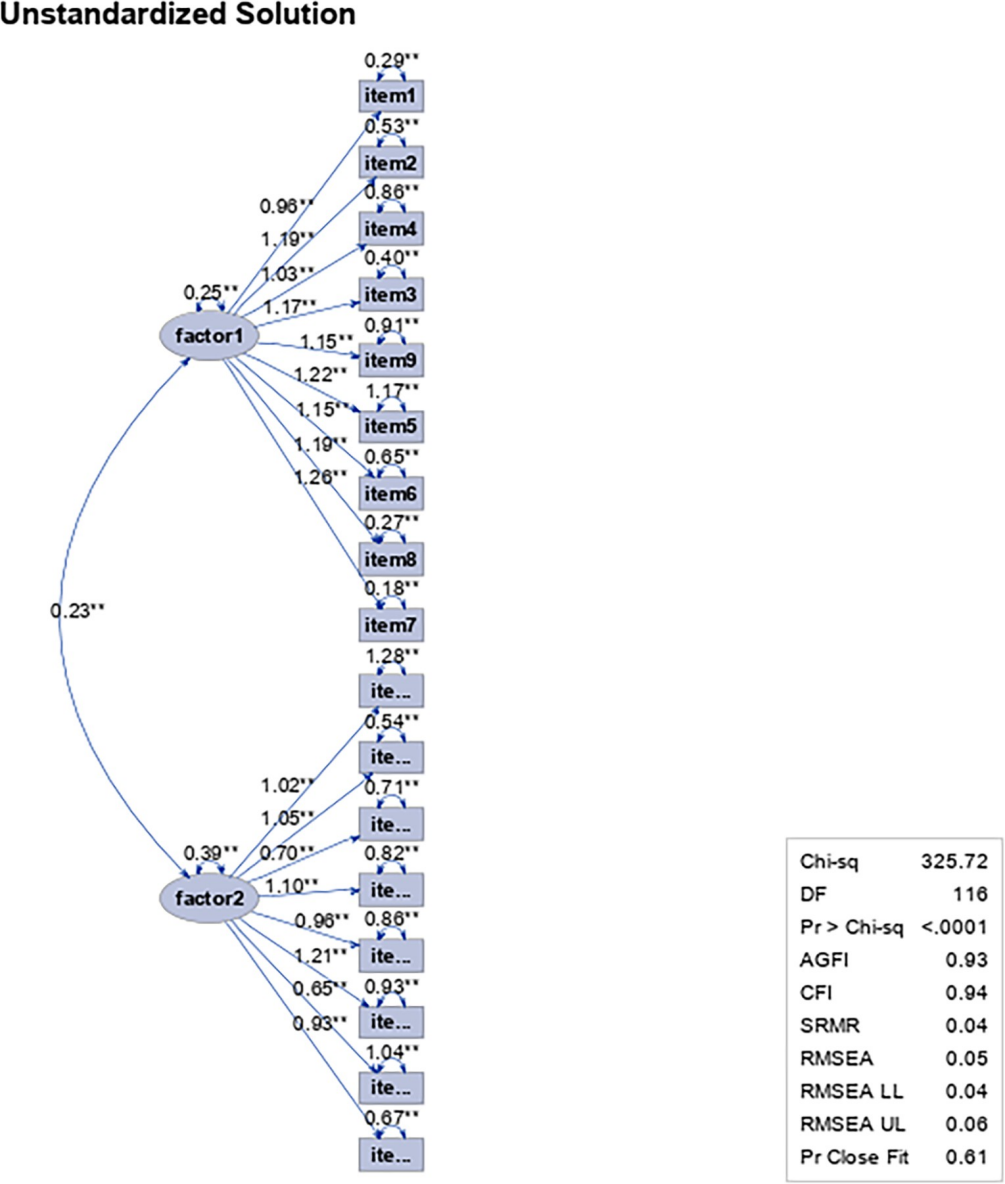
Path diagram for the first-order factorial analysis of the Leadership Virtues Questionnaire with 17 items and two factors in a sample of work and organizational psychologists and people who exercise leadership functions.

Based on the results obtained from the model adjustment indices, and since the model with four dimensions (first- and second-order analysis) shows an inefficient adjustment, as indicated above, an analysis of the model was conducted with two factors, which showed a marginally efficient adjustment (Fig 7).

Therefore, to decide the configuration of the dimensions, the complete model (four dimensions, original structure) was contrasted with the adjusted model (two dimensions, from the results obtained) according to the results of Table 6. An analysis of the comparison of the models for first- and second-order levels is performed.

**Table 6:** Statistical indices for the comparative analysis of the first- and second-order confirmation factors to evaluate the adjustment of the model in the Leadership Virtues Questionnaire in a sample of work and organizational psychologists and people who exercise leadership functions.

				Absolute Fit			Comparative Fit		Parsimonious Fit		ECVI
Model	Order	Factors	Items	χ^2^	υ	p	CFI	TLI	SRMR	RMSEA	
1	1	4	19	565.02	142	0.00	0.89	0.87	0.06	0.06	0.86
	2	4	19	1052.0	149	0.00	0.86	0.84	0.06	0.09	1.50
2	1	2	17	325.72	116	0.00	0.94	0.94	0.04	0.05	0.52
	2	2	17	676.65	117	0.00	0.91	0.90	0.05	0.08	0.99

See the legend of Table 4.

**Fig 8:**
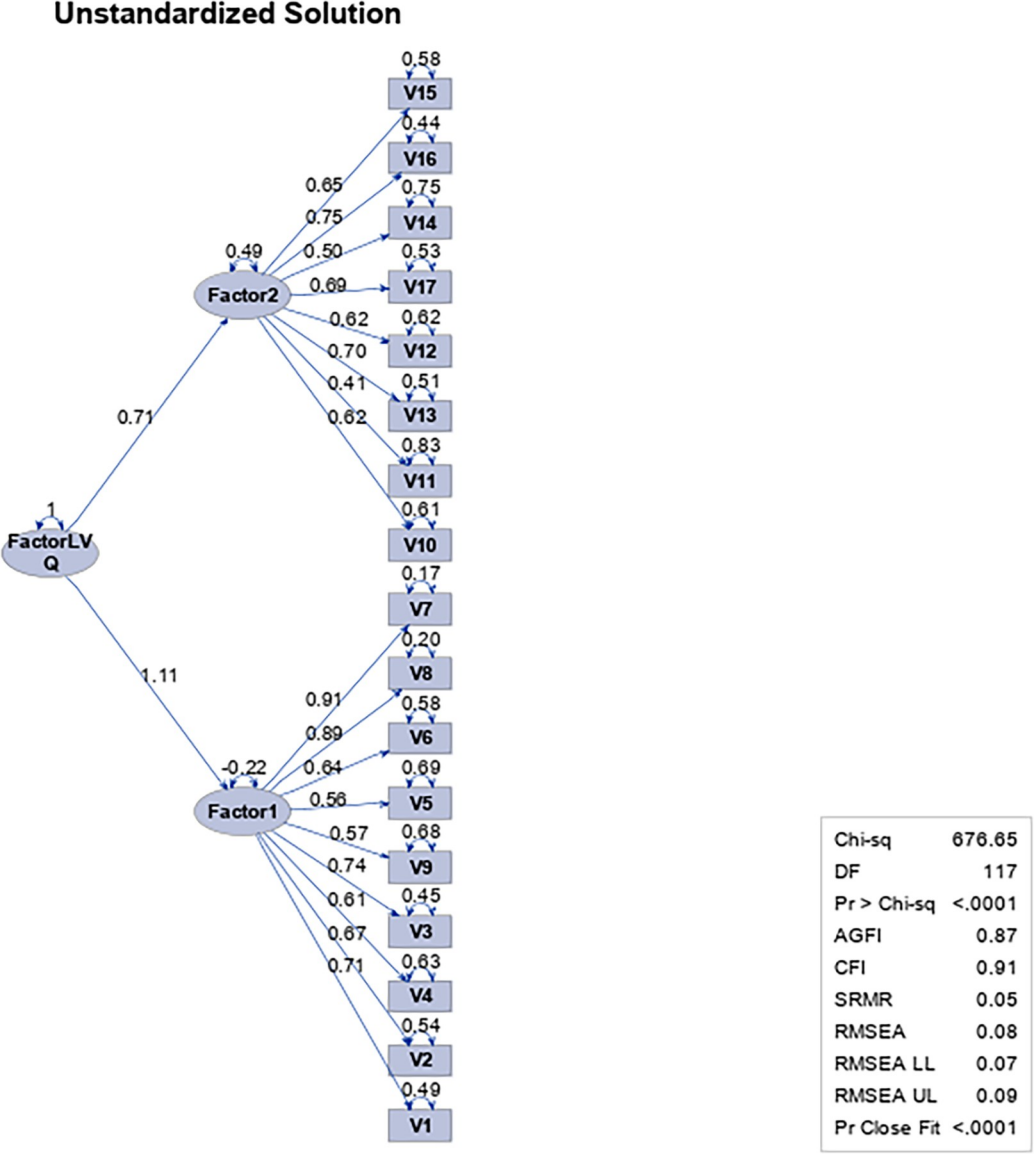
Path diagram for the second-order factorial analysis of the Leadership Virtues Questionnaire with 17 items and two factors in a sample of work and organizational psychologists and people who exercise leadership functions.

According to the values obtained, the first-order analysis of the complete model shows significant differences from the second-order analysis ☯χ^2^ (7) = 375.25, *p = 0*.*00*]. In turn, when comparing the complete model with the adjusted model (two dimensions), significant differences are observed only in the first-order analysis of the complete model (four dimensions) and two dimensions ☯χ^2^ (32) = 486.98, *p = 0*.*00*]. Consequently, since the adjusted model is efficient in absolute, comparative and parsimonious terms, it has been defined as methodological proposal to be used in this work (Fig 8).

## Discussion and conclusions

The objective of this work is to analyze the metric properties of the LVQ test scores when adapted to Spanish for work psychologists and organizations and applied in people who exercise leadership roles. This is conducted through an evaluation of the items and dimensions of prudence, justice, fortitude and temperance according to the parameters and the adjustment of the test model with the four original dimensions.

From the results obtained, 68% of the items have acceptable parameters, 10.53% have marginally acceptable parameters, and 21.05% exhibit insufficient values. Similarly, 83% of the correlations between the items are statistically significant, except for the conditions associated with items 12 and 13 (temperance dimension), while the correlations of the dimensions are equal to or less than 0.64.

Regarding metric properties, consistency is observed between the theoretical and empirical factor structures of the test, with final estimates of the explained variance of 98.71% (explaining 61.54, 13.70, 12.06, and 11.41, respectively). In turn, an acceptable global reliability (0.84) and marginally acceptable (between 0.74 and 0.77) is observed for the prudence and justice dimensions. However, they are insufficient (<0.66) for the dimensions of strength and temperance. In this context, the results are similar to those obtained by Riggio et al. [19] regarding the levels of reliability for the dimensions of prudence and justice.

In relation to the validity rates associated with dimensions, higher values in justice and prudence are observed. However, these cannot be compared to the work of Riggio et al. [19], as this does not report these indices.

Regarding the statistical indices for the first-order confirmatory factor analysis, the four-dimensional model has an inefficient adjustment, which behaves slightly better than the second-order model (although the indices are lower than those recommended in the literature empirical). As in the previous case, these results are lower for the comparative adjustment obtained by Riggio et al. [19].

In turn, using the parallel method, an adjusted configuration with two dimensions is observed. In this context, the scale retains 89.47% of the items (17 of 19, except for items 12 and 13) and shows a reconfiguration by factors with items 1,5,14,16,6,18,15,17, 11 (Factor 1, predominance of justice because it contains 5 of 9 items) as well as items 4,8,19,10,2,9,7,3 (Factor 2, predominance of strength because it contains 4 of 8 items). In this context, it should be noted that the items of the prudence dimension are distributed in the two dimensions and would support the virtues of fortitude and justice.

As for the items eliminated from the scale (12 and 13), first from the construction of the items, it is possible that the use of inverse meaning (recoding of responses) and of denials produce biases in the answers and affect the metric properties of the items (Suárez et al. [47]).

From the point of view of item content, in the temperance dimension (control of emotions), the wording of item 12 (with a denial at the beginning) could generate some difficulty in understanding the ethical behavior to be observed (careless personal achievements). While item 13 (*tends to observe a very vigilant management style*) would contradict the control of emotions.

With respect to the statistical analysis of the items, 21% show indices with low discrimination (correctly differentiate between low, moderate and high scores). This situation verified in parallel thanks to the observations made by people after responding to the test, which pointed out the difficulty in understanding some items. To solve this situation, for further studies, the original authors of the LVQ test (Riggio et al. [19]) were requested to make adjustments in the drafting of eleven items (57.89% of the test), which was supported by the main author (Riggio. Personal communication, April 25, 2023).

Despite the complexities described, from the metric point of view, recoded and denial items did not affect the values of statistical indices.

In relation to the contextual variables, Juyumaya [48] indicates that an argument to consider when evaluating the metric properties of an instrument in non-European populations is the influence of the country’s cultural characteristics in "organizational dynamics." In this regard, it should be noted that according to Hofstede [49] and Hofstede et al. [50], Chilean culture (according to model 6-D) is characterized in part by high indices of power distance (acceptance of unequal distribution of power in institutions and organizations), avoidance of uncertainty (development of beliefs and institutions to avoid threats of ambiguous or unknown situations) and indulgence (will to carry out their desires and impulses to enjoy life and have fun, giving greater importance to free time, among others). The latter could affect the responses to temperance items, while high values in the distance of power and avoidance of uncertainty may be related to the characteristics in which the work (control and surveillance) was given, affecting, in turn, the responses in the dimensions of justice (interpersonal relationships) and prudence (decision-making in situations of uncertainty).

In addition, in line with Pérez-Latre [8], elements that favor the exercise of leadership at the institutional level include confidence, the integration of rivals in work teams, participation in decision-making and dialogue as a more practical and reasonable solution to face leadership challenges (mental confusion, low creativity, insufficient communication skills, pessimism and laziness). In that context, possibly the responses of the items in the “Justice” dimension (virtue of social relations at the interpersonal and institutional level) could be influenced by the implications of situations of social violence that occurred in the country during 2019, a year marked for a deep crisis of trust, uncertainty and social discomfort with institutions.

Then, in early 2020, the forced installation of teleworking during the pandemic impacted organizational practices. It is noteworthy that since its inception, teleworking has been installed in an environment of distrust by employers, i.e., with fear of allowing their employees to work from their homes, far from the supervision of their direct supervisors (Bloom et al. [51]), partly for the loss of control (Tapasco-Alzate et al. [52]) when out of a physical space (Kirs et al. [53]), which may have been exacerbated during the pandemic and influenced the responses to temperance items (impoverished management of negative emotions) and prudence (due to low ability to make decisions with sufficient information).

The unhabitual teleworking conditions during this period and the increase in mental health problems, mainly depression and anxiety (Celis-Morales et al. [54]) could have affected behaviors associated with virtues in the leaders who supervised teleworkers as well as the workers themselves.

Considering the complexities of this time and space, the decision -making (prudence) processes of the leaders is very possibly at the basis of efforts to maintain moral behavior (strength) and administer fair resources (justice) during the time of pandemic.

At the same time, the application of the test in the context above described implied that, in some cases, an indirect record (realization of tasks without being in the usual or teleworking place) of the behaviors referred to in the items could be mediated by the perception or memory of pre-pandemic work situations.

Regarding the consistency found with other studies, although the statistical rates of reliability and validity have not been known to evaluate leadership based on virtues, based on the narrative description of the organization of the dimensions of the IES 360° test (Cardona et al. [35]), it is observed that the internal personal subdimension of self-government would have consistency with the virtues based on the competencies of decision-making (prudence), self-control (strength), emotional balance (temperance) and integrity (justice).

Similarly, there would be theoretical consistency of the virtue of temperance (Riggio et al. [19]) with the “commitment” dimension (Daniels et al. [10]) from the behavior of the follower (“vision”, as a behavioral ability to stay focused over time), the virtue of justice (“value” as an ethical behaviors promotion at all times) and the virtue of strength (“persistence” as a behavioral effort oriented to reinforcing contingencies of the environment).

However, for an adequate operationalization of virtue-based leadership from a competency-based approach or based on the impact of the leaders’ behavior on the followers, researchers or applied psychologists should consider the analysis criteria proposed by Luthans et al. [11], namely research (evidence of competent behaviors of leaders), strategy (key informants) and values (organizational culture), or operationalization of Cardona et al. [35], Daniels et al. [10] and Daniels et al. [30].

For example, based on the criteria of the studies (Luthans et al. [11]) and the findings of the present work, the LVQ test works from the point of view of the perception of third parties (see also Daniels et al. [10]), to guide the efforts to develop an objective and specific evaluation instead of being based on more general judgments (susceptible to cognitive, attentional and memory biases) that make it difficult to specify which are the specific behaviors corresponding to each virtue.

As for the definition of the construct, it would be appropriate to deliver guidelines that allow for improving management (Cardona et al. [35]) or the leadership exercise behavior model proposed by Daniels et al. [10]. Specifically, Cardona et al. [14] recommend that specific actions (behaviors) meet the conditions of measurable, affordable, relevant, specific and followable (Núñez [36]). An example in this context would be multifactorial leadership (Avolio et al. [15]).

*A fortiori*, in order to operationally address the virtues (or “integrity”, Daniels in Bailey et al. [24]) over time, it would be important for researchers to review the leadership model proposed by Daniels et al. [10] to guide changes to efficient management through the exercise of leadership skills through “background control (“vision”), the analysis of the task to achieve goals and the use of positive reinforcement in organizations in order to evaluate the impact that leader’s behaviors (model in leadership exercise) have on followers (Chapter 12 of Daniels et al. [10]). In addition, since the development of leadership is a process of accumulated work behaviors, it is important that, for the exercise of leadership, a coherent and consistent ethical code is used.

Finally, one advantage of these models would be their evidence based on evidence on learning behaviors, such as the ability to change the need for survival, and to face the growing demands that imply the development of new processes, as commonly observed in the field of organizational management. Furthermore, through this, the possibility of developing training programs aimed at optimal leadership profiles for the work space is enhanced. Therefore, as a training repertoire, leadership could be treated as an independent variable and influence the most ethical contingency organizations (Parks et al. [27]).

## Supporting information

S1 Appendix Spanish Version of Leadership Virtues. (DOCX)

## References

[1] Dye C. Leadership in Healthcare: Essential Values and Skills. 4^th^ ed. United States of America: ACHE Management Series; 2023.

[2] Regojo P. Ética para Directivos y Consejeros. Cómo Construir Empresas Excelentes y Socialmente Responsables. 1^st^ ed. España: EUNSA; 2014.

[3] Echevarría J. Dirigir Empresas con Sentido Cristiano. 3^th^ ed. España: EUNSA; 2017.

[4] Vilallonga M. Gestión del Talento y Desarrollo Organizativo. Algunas Claves. 1^st^ ed. España: EUNSA; 2020.

[5] Cardona P, Rey C. Management by Missions. Connecting People to Strategy Trough Purpose. 2^nd^ ed. Switzerland: Palgrave Macmillan; 2022.

[6] Ewest T. Leadership today. Practices for Personal and Professional Performance. Marques J, Dhidnam S, editores. 1^st^ ed. Switzerland: Springer; 2017.

[7] Sisón AJ. Liderazgo y Capital Moral. 1st ed. España: EUNSA; 2012

[8] Pérez-Latre F J. (2022). Crisis de Confianza (2007–2022) El Descrédito de los Medios. 1^st^ ed. España: EUNSA; 2022.

[9] Lussier R, Achua Ch. Liderazgo. Teoría, Aplicación y Desarrollo de Habilidades. 6^th^ ed. United States of America: Cengage Learning; 2016.

[10] Daniels A. Daniels J. Measure of a Leader. 1^st^ ed. United States of America: McGraw-Hill; 2007.

[11] Luthans F, Luthans BC, Luthans KW. Organizational Behavior. An Evidence- Based Approach. 14^th^ ed. United States of America: Information Age Publishing Inc. 2021

[12] Yukl G, Gardener W. Leadership in organizations. 3^th^ ed. United States of America: Pearson; 2020.

[13] Fuller M, Muñoz G, Lane A. Influential article review—redefining leadership principles. Journal of Leadership, Accountability and Ethics. 2019; 16 (6): 1–26.

[14] Cardona P, García-Lombardía P. Cómo Desarrollar las Competencias de Liderazgo. 1^st^ ed. España: EUNSA; 2011.

[15] Avolio B, Bass B. Multifactor Leadership Questionnaire. Manual and Sampler Set. 3tg ed. Redwood City: Mindgarden; 2004.

[16] Kibbe MR. Leadership Theories and Styles. En: Kibbe M, Chen H, editores. Leadership in Surgery. 2^nd^ ed. Switzerland: Springer; 2019. p. 27–36.

[17] Aasland MS, Skogstad A, Notelaers G, Nielsen MB, Einarsen S. The prevalence of destructive leadership behaviour. Br J Manag. 2009. doi: 10.1111/j.1467-8551.2009.00672.x

[18] Kumar RD, Khiljee N. Leadership in healthcare. Anaesthesia and Intensive Care Medicine. 2016 Jan 21; 17(1): 63–65. Available from: doi: 10.1016/j.mpaic.2015.10.012

[19] Riggio RE, Zhu W, Reina Ch, McKenna C, Maroosis J. Virtue-based measurement of ethical leadership: the leadership virtues questionnaire. Consulting Psychology Journal: Practice and Research. 2010 Dec 1; 62(4):235–250. doi: 10.1037/a0022286

[20] Fosse TH, Skogstad A, Einarsen SV, Martinussen M. Active and passive forms of destructive leadership in a military context: a systematic review and meta-analysis. Eur J Work Org Psychol. 2019;28(5):708–22. doi: 10.1080/1359432x.2019.1634550

[21] Reiche S, Bird A, Mendenhall M, Osland J. Contextualizing leadership: a typology of global leadership roles. J Int Bus Stud. 2017 Aug 02; 48:552–572. doi: 10.1057/s41267-016-0030-3

[22] Havard A. Perfil del líder. Hacia un Liderazgo Virtuoso. 1^st^ ed. España: Palabra S.A.; 2010.

[23] Havard A. Del Temperamento al Carácter. Cómo Convertirse en un Líder Virtuoso. 1^st^ ed. España: EUNSA; 2019.

[24] Bailey JS, Burch MR. Métodos de Investigación en Análisis Aplicado de Conducta, 2^nd^ ed. España: ABA España; 2021.

[25] Gadaire D, Kelley M, Larue R. Indirect Behavioral Assessments: Interviews and Rating Scales. In: Fisher W, Piazza C, Roane H, editores. Handbook of Applied Behavior Analysis. 2^nd^ ed. United States of America: Guilford Press; 2021. p. 193–201.

[26] Mattaini MA, Roose KM. Organization and Leadership in Resistance Movements. In Houmanfar RA, Fryling M, Alavasius M P Editors. Applied Behaviors Science in Organizations. 1^st^ Ed. United States of America: Routlege; 2022. p. 245–259.

[27] Parks N, Tudor A, Ventura A. Leadership in Behavior Analysis: The Independent Variable that Advances our Field. 1^st^ ed. United States of America: Behavior Leader Inc; 2022.

[28] Wilder D, Gravina N. Organizational Behavior Management. In: Fisher W, Piazza C, Roane H, editors. Handbook of Applied Behavior Analysis. 2^nd^ ed. United States of America: Guilford Press; 2021. p 544–558.

[29] Sarráis F. Psicopatología. 1^st^ ed. España: EUNSA; 2016.

[30] Daniels A, Bailey JS. Performance Management. Changing Behavior that Drives Organizational Effectiveness. 5^th^ ed. United States of America: Aubrey Daniels International Inc; 2014.

[31] American Psychiatric Association. The Diagnostic and Statistical Manual of Mental Disorders. Fifth Edition, Text Revision (DSM-5-TR™). United States of America: American Psychiatric Association; 2022.

[32] Koenig H. Religion and Mental Health: Research and Applications. 1^st^ ed. United States of America: Academic Press; 2018.

[33] Titus CS, Vitz PC, Nordling WJ, McWorther MR, Gross C. Fulfilled in Virtues. In: Vitz P, Craig ST, Nordling W; editors. A Catholic Christian Meta-Model of the Person: Integration with Psychology and Mental Health Practice. Divine Mercy University Press; 2020. p. 249–305

[34] Bass BM, Avolio BJ. MLQ Multifactor Leadership Questionnaire. Redwood City: Mind Garden; 2000.

[35] Cardona P, Wilkinson H. Creciendo como Líder. 1^st^ ed. España: EUNSA; 2009.

[36] Núñez A. El nuevo directivo público. Claves de Liderazgo para la Gestión Pública. 1^st^ ed. España: EUNSA; 2010.

[37] Norma UNE-ISO 10667. Prestación de servicios de evaluación. Procedimientos y métodos para la evaluación de personas en entornos laborales y organizacionales (partes 1 y 2). Madrid: AENOR; 2013.

[38] Livacic-Rojas P, Fernández P, Vallejo G, Tuero-Herrero E, Ordóñez F. Sensitivity of five information criteria to discriminate covariance structures with missing data in repeated measures designs. Psicothema. 2020 Aug; 32(3):399–409. doi: 10.7334/psicothema2020.63 32711676

[39] Ato M, Vallejo G. Diseños de Investigación en Psicología. 1^st^ ed. España: Pirámide; 2015.

[40] Muñiz J, Elosua P, Hambleton R. Directrices para la traducción y adaptación de los tests: segunda edición. Psicothema. 2013 May; 25(2): 151–157. doi: 10.7334/psicothema2013.24 23628527

[41] Bailey JS, Burch MR. 25 Habilidades Esenciales y Estrategias para Analistas de Conducta: Consejos de Expertos para ser Profesionales más Eficaces. 2^n^d España: ABA España; 2021.

[42] Institute SAS. SAS/SAT ® 14.3 User’s Guide. United States of America: SAS Institute Inc; 2017. Available from: https://support.sas.com/documentation/onlinedoc/stat/143/irt.pdf

[43] O’Rourke N, Hatcher L. A Step-by-Step Approach to Using SAS for Factor Analysis and Structural Equations Modeling. 2^nd^ ed. SAS Institute; 2013. Available from: https://sasinstitute.redshelf.com/book/1878387

[44] Pérez C. Regresión con Datos de Panel y Ecuaciones Estructurales: Ejercicios Con SAS, Eviews, Stata y SPSS. 1^st^ ed. United States of America: Createspace Independent Publishing Platform; 2016.

[45] Abad F, Olea J, Ponsoda V, García C. Medición en Ciencias Sociales y de la Salud I. 1^st^ ed. Madrid: Síntesis; 2011.

[46] Lind D, Marchal W, Wathen S. Estadística Aplicada a los Negocios y la Economía. 17^th^ ed. España: Mc Graw Hill; 2020.

[47] Suárez-Álvarez J, Pedrosa I, Lozano LM, García-Cueto E, Cuesta M, Muñiz J. Using reversed items in Likert scales: A questionable practice. Psicothema. 2018 May; 30(2): 149–158. doi: 10.7334/psicothema2018.33 29694314

[48] Juyumaya JE. Escala Utrecht de work engagement en Chile: Medición, confiabilidad y validez. Estudios de Administración. 2019;26(1):35–59. doi: 10.5354/0719-0816.2019.55405

[49] Hofstede G. Culture’s Consequences: Comparing Values, Behaviors, Institutions, and Organizations Across Nations. Thousand Oaks: Sage Publications; 2001.

[50] Hofstede G, Hofstede GJ, Minkov M. Cultures and Organizations: Software of the Mind. Revised and Expanded 3rd Edition. New York: McGraw-Hill; 2010.

[51] Bloom N, Liang J, Roberts J, Ying ZJ. Does working from home work? Evidence from a Chinese experiment. Q J Econ. 2015 Feb;130(1):165–218. doi: 10.1093/qje/qju032

[52] Tapasco-Alzate OA, Giraldo-García JA. Asociación entre posturas administrativas de directivos y su disposición hacia la adopción del teletrabajo. Información Tecnológica. 2020 Feb; 31(1):149–160. doi: 10.4067/S0718-07642020000100149

[53] Kirs PK. The impact of trust and changes in trust: a national comparison of individual adoptions of information and communication technologies and related phenomenon. International Journal of Information Management. 2012; 32:431–41. doi: 10.1016/j.ijinfomgt

[54] Celis-Morales C, Nazar G. Cambios en la prevalencia de depresión en Chile y el mundo debido a la pandemia por COVID-19. Rev Med Chil. 2022 May; 150(5):691–2. doi: 10.4067/s0034-98872022000500691 37906772

