# Supplementary material for: Validation and analysis of the metric properties of the Leadership Virtues Questionnaire in work and organizational psychologists and individuals who perform leadership functions in Chile
Source: PLoS One. 2024 Apr 18;19(4):e0297906. doi: 10.1371/journal.pone.0297906 (PMC11025730; doi:10.1371/journal.pone.0297906)
Supplement: S1 Appendix — (DOCX) [file pone.0297906.s001.docx]

**S1_Appendix Spanish Version of Leadership Virtues**

**Test de liderazgo basado en virtudes LVQ**

| Ítem | Afirmación | 1 | 2 | 3 | 4 | 5 |
| --- | --- | --- | --- | --- | --- | --- |
| 1 | Hace lo que debería en una situación determinada |  |  |  |  |  |
| 2 | No considera cuidadosamente toda la información disponible antes de tomar una decisión importante que impacte a otros. (R) |  |  |  |  |  |
| 3 | Se embarca audazmente en una situación sin considerar las consecuencias de sus acciones. (R). |  |  |  |  |  |
| 4 | No busca información de una variedad de fuentes de manera de tomar la mejor decisión. (R) |  |  |  |  |  |
| 5 | Considera un problema desde todos los ángulos y toma la mejor decisión para todas las partes involucradas. |  |  |  |  |  |
| 6 | Preferiría arriesgar su trabajo antes de cometer una injusticia. |  |  |  |  |  |
| 7 | Puede tener dificultad para defender sus creencias entre amigos que no comparten los mismos puntos de vista. (R) |  |  |  |  |  |
| 8 | No logra tomar la mejor decisión moral en una situación dada. (R) |  |  |  |  |  |
| 9 | Puede dudar en hacer cumplir normas éticas si se trata de un amigo cercano. (R) |  |  |  |  |  |
| 10 | Ignora su “voz interior” al decidir cómo proceder. (R) |  |  |  |  |  |
| 11 | Parece estar demasiado preocupado de su poder personal. (R) |  |  |  |  |  |
| 12 | No está demasiado preocupado de sus logros personales. |  |  |  |  |  |
| 13 | Desea saber todo lo que sucede en la organización hasta el punto de realizar una detallada supervisión (microgestión). |  |  |  |  |  |
| 14 | Da crédito a otros cuando es debido |  |  |  |  |  |
| 15 | Demuestra respeto por todas las personas. |  |  |  |  |  |
| 16 | Puede atribuirse el crédito de los logros de otros. (R) |  |  |  |  |  |
| 17 | Respeta los derechos e integridad de los demás. |  |  |  |  |  |
| 18 | Podría tomar decisiones de promoción basadas en el mérito de un candidato. |  |  |  |  |  |
| 19 | No trata a los demás como le gustaría ser tratado. (R) |  |  |  |  |  |

R= ítem invertido. Escala de respuestas: 1= en lo absoluto; 2= de vez en cuando; 3= a veces; 4= con bastante frecuencia; 5= con frecuencia, aunque no siempre.

**Dimensión Prudencia** *(toma de decisiones):* Ítems 1 al 5;

**Dimensión Fortaleza** *(manejo del estado del ánimo):* Ítems 6-10;

**Dimensión Templanza** *(autocontrol personal)*: Ítems 11-13;

**Dimensión Justicia** (*relaciones interpersonales*): Ítems 14 al 19.
